# Black-backed jackals (*Canis mesomelas*) are natural hosts of *Babesia rossi*, the virulent causative agent of canine babesiosis in sub-Saharan Africa

**DOI:** 10.1186/s13071-017-2057-0

**Published:** 2017-03-13

**Authors:** Barend L. Penzhorn, Ilse Vorster, Robert F. Harrison-White, Marinda C. Oosthuizen

**Affiliations:** 1 Department of Veterinary Tropical Diseases, Faculty of Veterinary Science, University of Pretoria, Private Bag X04, Onderstepoort, 0110 South Africa; 2 Research Associate, National Zoological Gardens, Boom Street, Pretoria, South Africa; 3 Wildlife Damage - Research and Management, P.O. Box 783540, Sandton, 2146 South Africa

**Keywords:** *Babesia rossi*, Black-backed jackal, *Canis mesomelas*, Natural host, South Africa

## Abstract

**Background:**

*Babesia rossi*, which is transmitted by *Haemaphysalis* spp. and is highly virulent to domestic dogs, occurs only in sub-Saharan Africa. Since dogs are not native to the region, it has been postulated that the natural host of *B. rossi* is an indigenous African canid. Although various attempts at artificial infection indicated that black-backed jackals (*Canis mesomelas*) could become subclinically infected with *B. rossi*, data on occurrence of *B. rossi* in free-ranging jackals was lacking. A long-term behaviour study in which free-ranging black-backed jackals were radio-collared offered the opportunity of collecting blood specimens from a large number of free-ranging jackals.

**Methods:**

Genomic DNA was extracted from the EDTA blood samples (*n* = 107). PCR products were subjected to Reverse Line Blot hybridization using *Theileria* and *Babesia* genera-specific as well as 28 species-specific oligonucleotide probes, including *Babesia canis*, *Babesia rossi*, *Babesia vogeli* and *Babesia gibsoni*. The near full-length parasite 18S rRNA gene was amplified from two selected samples (free-ranging jackals), cloned and a total of six recombinants were sequenced.

**Results:**

Of 91 free-ranging jackals, 77 (84.6%) reacted with the *Babesia* genus-specific probe; 27 (29.7%) also reacted with the *B. rossi* probe. Of 16 captive jackals, 6 (37.5%) reacted with the *B. rossi* probe, while one further sample reacted with the *Babesia* genus-specific probe only. After cloning, 6 recombinants yielded identical sequences identical to that of *B. rossi* (L19079) and differing by 2 base pairs from *B. rossi* (DQ111760) in GenBank. The observed sequence similarities were confirmed by phylogenetic analyses using neighbour joining and maximum parsimony.

**Conclusions:**

Black-backed jackals are natural hosts of *B. rossi.*

## Background

Canine babesiosis remains a major concern over large parts of South Africa [1]. The main causative organism, *Babesia rossi*, has only been reported from sub-Saharan Africa. The less virulent *Babesia canis* (*sensu stricto*), transmitted by *Dermacentor reticulatus*, is restricted to Europe, while the least virulent *Babesia vogeli*, transmitted by *Rhipicephalus sanguineus* (*sensu lato*), has a cosmopolitan distribution that includes sub-Saharan Africa. Female *Haemaphysalis elliptica* (and presumably also *Haemaphysalis leachi* [2]) ticks, having become infected with *B. rossi* when engorging on infected hosts, transmit the infection transovarially. Larvae of the next generation and nymphs remain infected while engorging primarily on small rodents; *B. rossi* is transmitted when next-generation adults engorge, primarily on carnivores [3, 4].

Domestic dogs have been present for centuries or even millennia in sub-Saharan Africa, but they are not native to the region [5]. Since *B. rossi* and its vector(s) only occur here, Penzhorn [6] postulated that at least one canid indigenous to sub-Saharan Africa, e.g. a jackal or the African wild dog (*Lycaon pictus*), is a natural host of *B. rossi*.

Soon after babesiosis (malignant jaundice or bilious fever) was reported from domestic dogs in the Cape Colony, South Africa, in the 1890s [7] there were two attempts at transmitting infection from domestic dogs to black-backed jackals (*Canis mesomelas*), both by tick-feeding and blood inoculation. These attempts were not successful [8, 9] leading to a conclusion that the jackals were “quite immune” and probably not involved in maintenance of the infection in nature.

In 1947 Neitz & Steyn [10] established *B. rossi* infection in one spleen-intact and two asplenic black-backed jackals. Apart from mild anaemia and icterus, no other clinical signs were observed in the spleen-intact jackal and it made an uneventful recovery. The two asplenic jackals developed severe anaemia and icterus, but recovered despite not being treated. Piroplasms were still present three years later, when pooled blood from all three jackals injected intravenously into two domestic dogs resulted in fatal babesiosis. In a later study four black-backed jackal pups were inoculated intravenously with blood from a dog in the terminal stages of babesiosis [11]. All jackals developed parasitaemia not exceeding 0.04%, but showed no other clinical or haematological evidence of babesiosis. Domestic dog pups subinoculated with blood from these jackals all succumbed to babesiosis. These two studies demonstrated that the jackals could become subclinical carriers of *B. rossi*, which suggested that they may be natural hosts, but data from free-ranging natural jackal populations were still lacking.

A Predator Biodiversity Project aimed at developing alternative and more ecologically friendly strategies for effective and practical management of problem carnivores, including black-backed jackals, was launched in 1998. The project focuses on the social behaviour of problem carnivores, specifically with respect to their impact on prey animals and the factors influencing prey selection and population density. Since 2011, blood samples taken routinely whenever study animals are handled, e.g. for fitting radio collars, have been sent to the Department of Veterinary Tropical Diseases (DVTD), University of Pretoria (UP), for diagnostic purposes. This offered the first opportunity for determining whether *B. rossi* occurs in a free-ranging black-backed jackal population.

## Methods

### Sample collection

The main study site was the 3 068-hectare Mogale’s Gate Biodiversity Centre (25.9307°S, 27.6425°E) comprising mixed bushveld on the southern slopes of the Witwatersberg at border between North West Province and Gauteng Province, South Africa. Jackals were darted and immobilised by intramuscular injection of a combination of tiletamine and zolazepam (Zoletil®, Virbac Animal Health, Halfway House, South Africa). Blood was collected from the cephalic vein into EDTA tubes. Specimens were frozen and transported from the field to the Molecular Biology Laboratory, DVTD, UP, for further processing. For comparative purposes, blood specimens were also collected from black-backed jackals kept in large enclosures at S.A. Lombard Nature Reserve, 17 km northwest of Bloemhof (27.6263°S, 25.5800°E), North West Province, South Africa.

### DNA extraction

Genomic DNA was extracted from the EDTA blood samples (*n* = 107) using the QIAamp® DNA Mini Kit (Qiagen, Southern Cross Biotechnology, Cape Town, South Africa) according to the manufacturer’s instructions. DNA was eluted in 100 μl elution buffer and stored at -20 °C.

### Reverse Line Blot (RLB) hybridisation

The RLB hybridisation assay was performed as previously described [12–14]. Primers RLB F2 (5′-GAC ACA GGG AGG TAG TGA CAA G-3′) and biotin-labelled RLB R2 (5′-Biotin-CTA AGA ATT TCA CCT CTA ACA GT-3′) [14] were used to amplify the V4 hypervariable region of the *Theileria* and *Babesia* 18S rRNA gene. Platinum® Quantitative PCR SuperMix-UDG (LTC Tech SA, Johannesburg, South Africa) was used to perform the PCR following a touchdown thermal cycling program [14]. *Babesia bovis* DNA extracted from the *B. bovis* vaccine (Onderstepoort Biological Products, Tshwane, South Africa), were used as a positive control and water was used as the negative control. PCR products were subjected to the RLB hybridization as described by Nijhof et al. [14] using *Theileria* and *Babesia* genera-specific as well as 28 species-specific oligonucleotide probes, including *B. canis* [15], *B. rossi* [15], *B. vogeli* [15] and *Babesia gibsoni* [13].

### 18S rRNA amplification, cloning and sequencing

The near full-length 18S rRNA gene (1,700 bp) of two jackal specimens, that tested positive for *B. rossi* on the RLB assay, were amplified using Nbab_1F (5′-AAG CCA TGC ATG TCT AAG TAT AAG CTT TT-3′) and TB_Rev (5′-AAT AAT TCA CCG GAT CAC TCG-3′) [16, 17]. High Fidelity PCR Master Mix (Roche Diagnostics, Mannheim, Germany) was used to perform the PCR. Five separate reactions were prepared per sample; amplicons of all five reactions per sample were pooled to avoid Taq polymerase-induced errors and cleaned-up using the High Pure PCR Product Purification Kit (Roche Diagnostics, Mannheim, Germany) prior to cloning.

Using the pGEM-T Easy Vector system (Promega, Madison, WI, USA), the purified PCR fragment was ligated into the pGEM-T Easy vector and transformed into competent *E. coli* JM109 cells (JM109 High Efficiency Competent Cells, Promega, Madison, WI, USA). Colonies were picked and grown in imMedia Amp Liquid broth (LTC Tech SA, Johannesburg, South Africa) where after isolation of the recombinant plasmids was done using the High Pure Plasmid Isolation Kit (Roche Diagnostics, Mannheim, Germany). Sequencing was performed at Inqaba Biotec™ (Pretoria, South Africa) using the vector primers SP6 (5′-TTA TAC GAC TCA CTA TAG GG-3′) and T7 (5′-TAT TTA GGT GAC ACT ATA-3′).

The obtained sequences were assembled and edited using the GAP4 program of the Staden package (version 1.6.0 for Windows) [18]. Homologous sequence searches of databases were performed using the BLASTn package [19]. A multiple sequence alignment was performed using ClustalX (version 1.81 for Windows) which included all related available genera from GenBank [20]. The alignment was truncated to the size of the smallest sequence (1,513 bp) using BioEdit v7 [21]. Similarity matrices were constructed from the aligned sequence data by single distance, using the two-parameter model of Kimura [22]. The Jukes and Cantor correction model [23] was applied for multiple base changes. Phylogenetic trees were constructed using MEGA7 [24] using both neighbour joining [25] and maximum parsimony. Bootstrapping was applied using 1,000 replicates/trees for the distance method and 100 replicates/trees for the parsimony method [26]. All consensus trees generated were edited using MEGA7 [24].

The 18S rRNA gene sequences of the sequences identified in this study were submitted to GenBank (KY463429–KY463434).

## Results

The results of the RLB hybridization are shown in Table 1. Of the 91 free-ranging jackals, 77 (84.6%) reacted with the *Babesia* genus-specific probe; 27 (29.7%) also reacted with the *B. rossi* probe. Of the 16 captive jackals, 6 (37.5%) reacted with the *B. rossi* probe, while a further sample reacted with the *Babesia* genus-specific probe only.

**Table 1:** Prevalence of *Babesia rossi* in black-backed jackals at two collection sites, as determined by reverse line blot hybridisation assay

Locality	Total	*B. rossi-*positive	Genus-specific probe positive	
			T/B^a^	*Babesia1*
Mogale’s Gate	91	27	30	77
SALNR^b^	16	6	7	7
	107	33	37	84

^a^ *Theileria/Babesia*

^b^SA Lombard Nature Reserve

To confirm the *B. rossi* RLB results, the near full-length parasite 18S rRNA gene was amplified from 2 selected samples (free-ranging jackals), cloned and a total of 6 recombinants were sequenced. The resulting sequences were identical (1,513 bp), indicating a single infection. BLASTn homology search results revealed no identical sequences in the public databases. The most closely related sequence, with approximately 99% identity was *B. canis rossi* (GenBank L19079 and DQ111760).

**Fig. 1:**
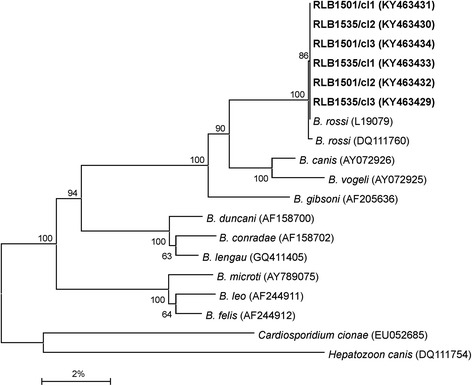
A neighbour-joining tree, with the Kimura two-parameter distance calculation, showing the phylogenetic relationship of the obtained sequences from piroplasms from jackals to related species based on the near full-length 18S rRNA gene sequences. *Hepatozoon canis* (DQ111754) and *Cardiosporidium cionae* (EU052685) were used as outgroup

A comparison of estimated evolutionary divergence between the observed gene sequences and published *B. rossi*, *B. canis*, *B. vogeli* and *B. gibsoni* 18S rRNA gene sequences was subsequently compared by determining the number of base differences per near full-length 18S rRNA gene sequence. All positions containing gaps and missing data were eliminated. There were a total of 1,502 positions in the final dataset. The obtained recombinant sequences were identical to that of *B. rossi* (L19079) and differed by two base pairs from *B. rossi* (DQ111760). It furthermore differed by 64, 73 and 76 base pairs from *B. canis* (AY072926), *B. vogeli* (AY072925) and *B. gibsoni* (AF205636), respectively. The observed sequence similarities were confirmed by phylogenetic analyses using neighbour joining and maximum parsimony techniques; no significant changes in the topology of the trees or in the bootstrap values were found. A representative tree obtained by the neighbour-joining method is shown in Fig. 1. The obtained sequences formed a monophyletic group with the published *B. rossi* sequences which in turn formed a monophyletic group with *B. canis*, *B. vogeli* and *B. gibsoni*.

## Discussion

*Babesia* spp. infections were common among the free-ranging jackals, as indicated by 77 of the 91 samples (84.6%) reacting with the *Babesia* genus-specific probe on RLB. Nearly one third of all jackals were infected with *B. rossi*, as confirmed by sequence analysis. The situation was virtually the same in the smaller captive population (*n* = 16), with 6 jackals (37.5%) being infected with *B. rossi*. Since previous studies [10, 11] indicated that *B. rossi* can become established in black-backed jackals without overt clinical signs developing, it is fair to assume that the positively reacting jackals in our study were subclinical carriers of *B. rossi*.

It will be interesting to determine to what extent other black-backed jackal populations are infected with *B. rossi*. Black-backed jackals occur in two discrete geographical ranges, separated by 900 km: north-east Africa (Somalia and eastern Ethiopia southward to Tanzania) and south-western Africa (from south-western Angola and Zimbabwe to the Western Cape Province, South Africa) [27]. Since *H. elliptica*, the known vector, prefers more mesic habitats [28], local jackal populations in arid and semi-arid areas are probably not infected. In southern Africa this would include the Karoo, Kalahari and most of the Atlantic coastline.

Our findings do not rule out the possible involvement of further natural hosts. The side-striped jackal (*Canis adustus*) from which *B. rossi* was initially described and named is a prime candidate [29, 30], but confirmatory data are lacking. Side-striped jackals are also widely distributed in sub-Saharan Africa: from Northern Nigeria eastward to south-western Ethiopia, southwards to the north-eastern parts of South Africa and westwards through Zimbabwe and Zambia into Angola [31]. There is large overlap between the distribution ranges of black-backed and side-striped jackals. A third potential natural host is the Ethiopian golden wolf (*Canis anthus*), previously regarded as the same species as the golden jackal (*Canis aureus*) of the Middle East, Eastern Europe and Asia [32]. In East Africa its distribution overlaps with that of both black-backed and side-striped jackals.

Domestic dogs do not occur at Mogale’s Gate Biodiversity Centre, our main study site, but the black-backed jackals move freely between Mogale’s Gate and surrounding farming areas where domestic dogs are kept. Tick transfer of infection between domestic dogs and jackals cannot be ruled out. *Babesia rossi* occurs as various genotypes and it was suggested that different *B. rossi* Br*EMA1* genotypes may cause differing host responses to infection (i.e. there could be a relationship between parasite genotypes and disease pathogenesis) [33]. The question arises whether *B. rossi* genotypes are host-specific, i.e. whether they are common to both jackals and dogs, or whether some occur only in one host but not the other. Genotypes occurring only in the jackals may represent highly virulent ancestral types that never became established in dog populations. Genotypes commonly occurring in dogs, on the other hand, may represent types that have evolved to be less virulent. Determination and characterisation of genotypes occurring in the jackal population may shed light on this issue.

## Conclusions

Two previous artificial-transmission studies demonstrated that *B. rossi* can become established in black-backed jackals without causing overt clinical signs, i.e. that the jackals became sub-clinical carriers of the piroplasm. Our study showed that *B. rossi* occurred frequently in a free-ranging black-backed jackal population. We therefore conclude that black-backed jackals are natural hosts of *B. rossi*.

## Abbreviations

DAFF: Department of agriculture forestry and fisheries South Africa
DVTD, UP: Department of veterinary tropical diseases University of Pretoria
EDTA: Ethylenediaminetetraacetic acid
RLB: Reverse line blot
